# The role of cytokines in the pathogenesis of SAPHO syndrome

**DOI:** 10.3389/fimmu.2024.1427784

**Published:** 2024-09-02

**Authors:** Yi Yang, Qianzhu Chen, Weiyang Zhong

**Affiliations:** ^1^ Department of Orthopaedic Surgery, The First Affiliated Hospital of Chongqing Medical University, Chongqing, China; ^2^ Orthopedic Laboratory of Chongqing Medical University, Chongqing, China

**Keywords:** SAPHO syndrome, cytokines, TNF-α, IL-1β, inflammatory response, immune regulation

## Abstract

SAPHO syndrome is a complex inflammatory disorder affecting the skin and bones, characterized by osteomyelitis, acne, and pustulosis. Cytokines play a pivotal role in the pathogenesis of SAPHO syndrome, especially in inflammatory responses and immune regulation. This article reviews the cytokines involved in the pathogenesis of SAPHO syndrome, such as tumor necrosis factor α (TNF-α), interleukin 1β (IL-1β), IL-6, IL-10, and transforming growth factor-β (TGF-β), and discusses their potential as intervention points for treatment. These findings elucidate the intricate immune regulatory network of SAPHO syndrome and provide a theoretical foundation for the development of new targeted therapeutic strategies.

## 1 Introduction

SAPHO syndrome, a rare immune-mediated disorder, is characterized by its primary clinical manifestations: synovitis (inflammation of the synovial membrane, leading to joint pain and swelling), acne (severe acneiform eruptions, often resistant to conventional treatments), pustulosis (pustular skin lesions, commonly affecting the palms and soles), hyperostosis (excessive bone growth, particularly in the sternoclavicular region), and osteitis (inflammation of the bone, causing pain and structural changes), from which its acronym is derived. First described in 1987, the syndrome encapsulates a spectrum of diseases sharing similar clinical and radiological features (1). The clinical manifestations of SAPHO syndrome are diverse, ranging from dermatological symptoms like severe acne and pustulosis to complex osteoarticular symptoms including pain and swelling in the sternum, clavicle, and spine (2, 3).

SAPHO syndrome can affect individuals of any age, but it typically presents in young adults (4). There is no strong sex predilection, although some studies suggest a slight female predominance. The prevalence of SAPHO syndrome varies by region, with higher reporting rates in Europe and East Asia, potentially reflecting differences in diagnostic practices and awareness (5). The clinical course of SAPHO syndrome is often chronic and relapsing, significantly impacting patients’ quality of life. The dermatological and osteoarticular manifestations are frequently the most debilitating, necessitating a multidisciplinary approach to management (6).

Despite advancements in SAPHO syndrome research over the past decades, the precise etiology and pathogenesis of the disorder remain elusive. It is currently hypothesized that SAPHO syndrome could be linked to a variety of factors, including genetic predispositions, immune system abnormalities, and microbial infections. The role of cytokines in its pathogenesis is particularly emphasized, as these key signaling molecules regulate immune and inflammatory responses. Understanding their impact is critical for elucidating the mechanisms of the disease. Moreover, since existing treatments for SAPHO syndrome primarily address symptoms and lack targeted therapeutic approaches, developing novel treatments based on cytokine modulation is of paramount importance.

This review article aims to thoroughly examine the central role of cytokines in the pathogenesis of SAPHO syndrome. Pro-inflammatory cytokines like tumor necrosis factor α (TNF-α) and interleukin 1β (IL-1β) are crucial in promoting inflammatory responses, exacerbating tissue damage and pain by activating pathways such as nuclear factor κB (NF-κB). Conversely, anti-inflammatory cytokines such as IL-10 and transforming growth factor β (TGF-β) strive to restore immune balance by mitigating inflammatory responses. This discussion of cytokine involvement not only provides a theoretical basis for new targeted therapies but also explores directions for future treatment improvements to enhance clinical outcomes.

## 2 SAPHO syndrome

### 2.1 Pathophysiology of SAPHO syndrome

SAPHO syndrome encompasses a spectrum of inflammatory skin and skeletal disorders, primarily characterized by bone proliferation and osteitis. Commonly, these bone alterations manifest in the anterior chest wall, including the sternum and clavicle, and extend to the cervical vertebrae and sacroiliac joints. Typically, the lesions involve bone sclerosis and hypertrophy of the adjacent soft tissues (7). Pathologically, bone lesions in SAPHO syndrome may present as acute inflammatory responses, and as the condition progresses, bone marrow fibrosis and significant sclerosis of bone trabeculae may develop (8). These features distinguish SAPHO syndrome from other forms of osteitis, such as infectious osteitis (3, 9).

The dermatological manifestations of SAPHO syndrome vary, with the most prevalent being severe acne and pustular eczema, especially palmoplantar pustulosis. These skin lesions can manifest either before or concurrently with the skeletal symptoms (10). Skeletal symptoms typically include pain and swelling in the anterior chest wall and chronic pain, often associated with bone proliferation and inflammation near joints. This bone proliferation can impair the function of adjacent joints, adversely affecting the patient’s quality of life (3, 11, 12). Imaging examinations are vital for diagnosis, revealing characteristic changes in the affected bones, including bone proliferation and erosion (13).

### 2.2 Pathogenesis

SAPHO syndrome is an immune-mediated inflammatory disorder whose pathogenesis involves complex interactions between genetic and environmental factors. Although specific genetic markers have not been conclusively identified, genetic susceptibility plays a crucial role in SAPHO syndrome. Research has indicated that particular genetic variations, such as mutations in the PSTPIP2 and NOD2 genes, might be linked to the onset of SAPHO syndrome; however, these findings necessitate further confirmation (14). Environmental factors, especially infectious agents, are considered significant triggers for the syndrome. Notably, infection with *Propionibacterium acnes* is believed to potentially trigger inflammatory responses by activating the immune system (15–17).

The immune response in SAPHO syndrome is characterized by both autoimmune and inflammatory components. Studies show that the immune responses, particularly those mediated by neutrophils and T cells, are overly active in affected individuals. For instance, neutrophils in patients with SAPHO syndrome demonstrate excessive activation, releasing substantial quantities of pro-inflammatory cytokines like IL-8 and TNF-α (17). Furthermore, the signaling pathways involving IL-1 and TNF-α are central to the pathophysiology of SAPHO syndrome, supporting the use of biologic treatments such as TNF-α inhibitors and IL-1 receptor antagonists (6, 10, 18).

## 3 Biological basis of cytokines

### 3.1 Definition of cytokines

Cytokines are a class of small protein molecules secreted by cells, primarily involved in signal transmission between cells to regulate immune and inflammatory responses. They interact with specific cell surface receptors and modulate the differentiation, migration, and activation of immune cells. This class includes various types such as interleukins (ILs), tumor necrosis factors (TNFs), and interferons (IFNs), which are pivotal in both innate and adaptive immunity. Given the extensive bioactivity and overlapping functions of cytokines, a detailed biological understanding is essential for the development of therapeutic strategies against diverse diseases (7, 19, 20).

### 3.2 Cytokines and the immune system

Cytokines play a pivotal role in immune regulation by modulating the activities of various immune cells, such as T cells, B cells, and macrophages, thereby shaping the body’s response to pathogens. For example, IL-2, a crucial growth factor, is essential for the proliferation and survival of T cells, while TNF and IL-6 are key in initiating and sustaining inflammatory responses. Moreover, cytokines influence cell migration and localization within tissues, enhancing the efficiency and precision of immune responses (21). Recent studies have also shown that cytokines such as IL-10 and TGF-β possess anti-inflammatory properties that regulate immune responses, thus mitigating excessive immune activity and preventing damage to host tissues (22–24).

## 4 Role of cytokines in the SAPHO syndrome

### 4.1 TNF-α

**Figure 1:**
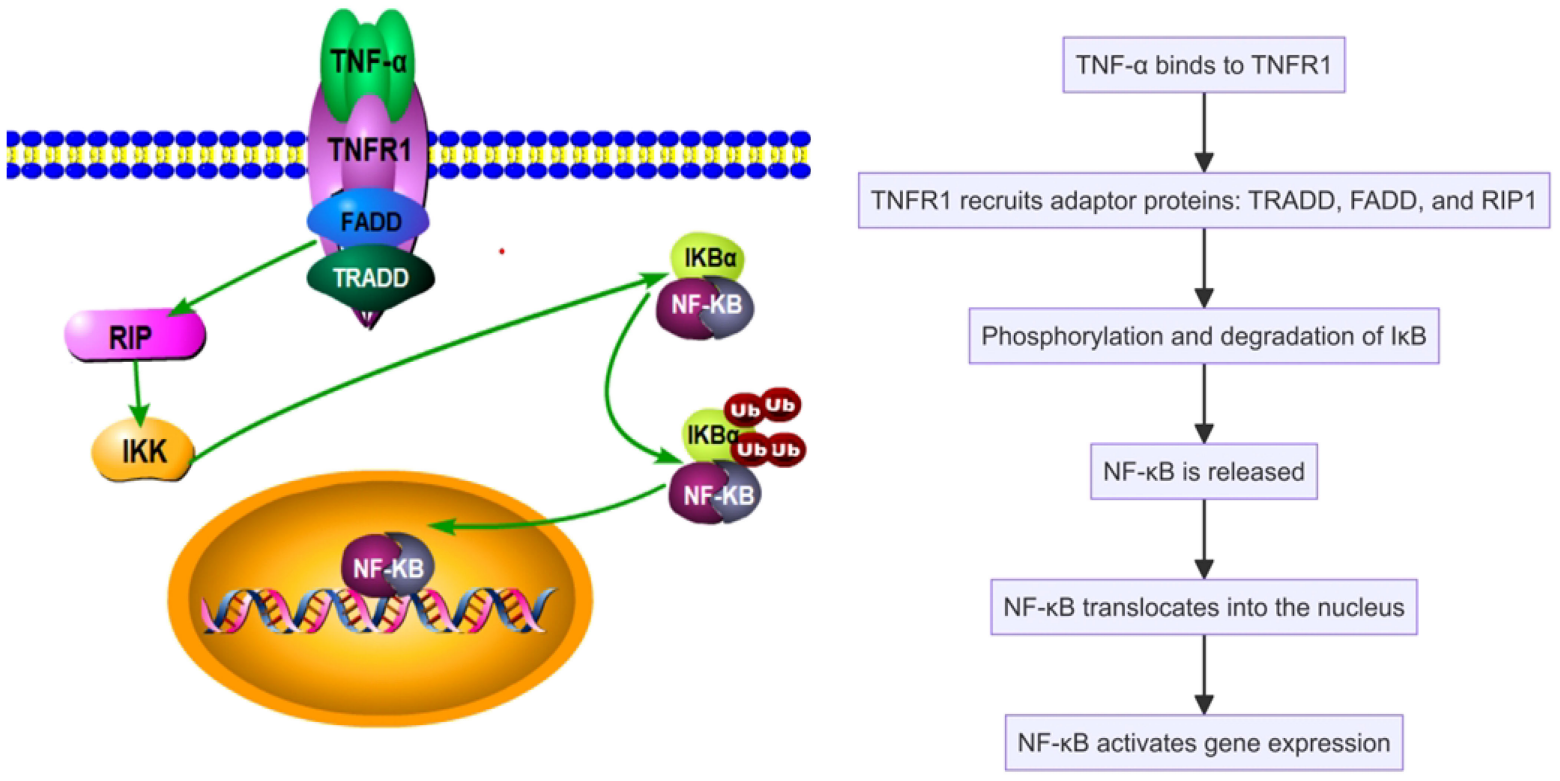
Tumor necrosis factor α (TNF-α) activates the nuclear factor κB (NF-κB) pathway in the SAPHO syndrome.

**Figure 2:**
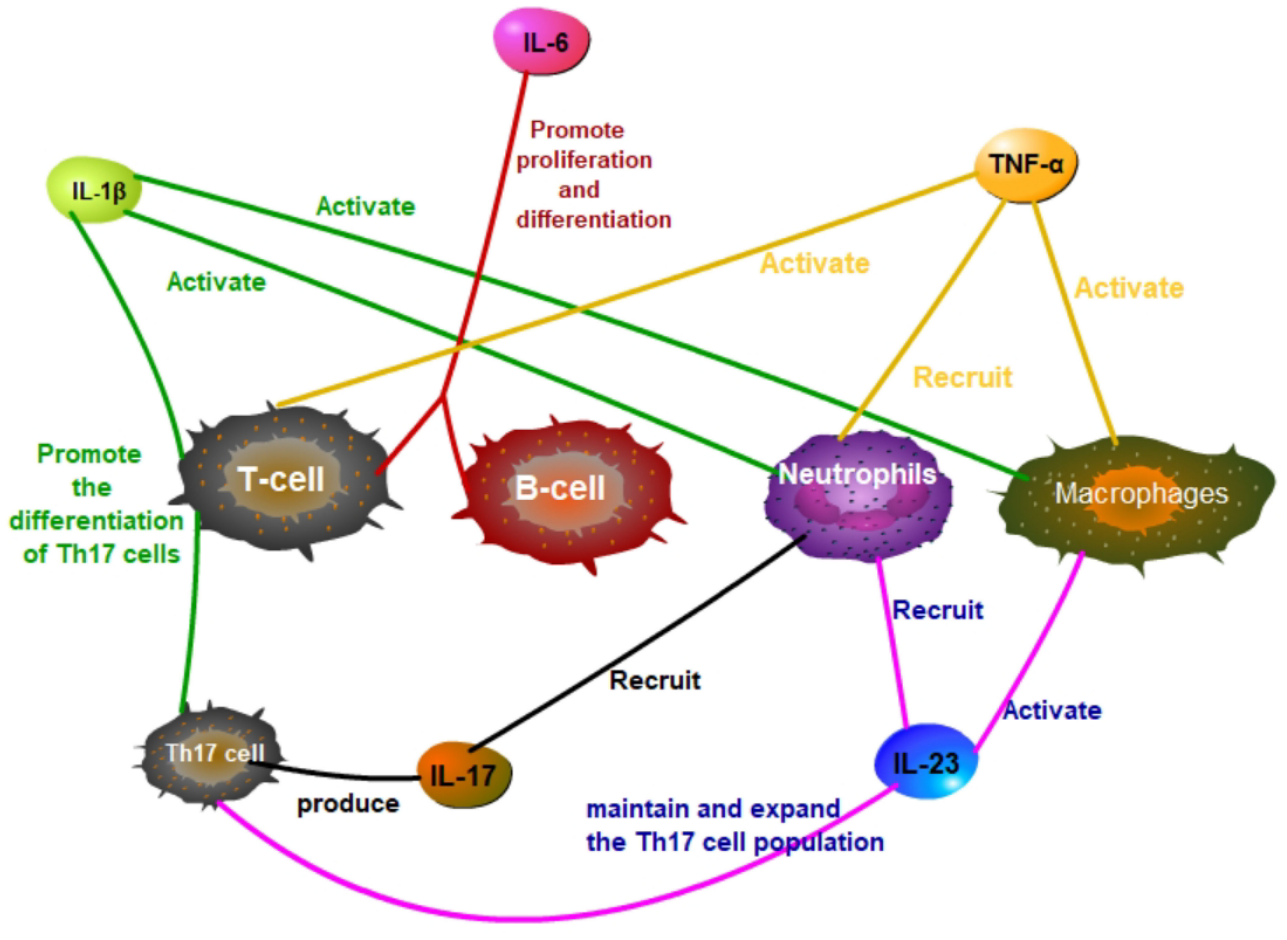
In SAPHO syndrome, IL-1β, IL-6, TNF-α, IL-17, and IL-23 have significant effects on T cells, B cells, macrophages, and neutrophils.

In SAPHO syndrome, tumor necrosis factor α (TNF-α) serves as a key pro-inflammatory cytokine, significantly influencing inflammatory responses and bone remodeling. TNF-α regulates inflammation, immune responses, and cell survival by activating cellular signaling pathways, notably the nuclear factor κB (NF-κB) pathway (25). TNF-α operates via its receptors, TNFR1 and TNFR2, with TNFR1 activation being particularly critical. Upon binding, TNFR1 initiates the formation of a signaling complex by recruiting adaptor proteins such as TRADD, FADD, and RIP1. This complex activation leads to the phosphorylation and degradation of IκB, consequently releasing NF-κB to translocate into the nucleus and activate gen expression related to inflammation, cell proliferation, and survival (**Figure 1**). TNF-α also drives inflammatory and cellular responses by stimulating the MAPK pathway, which includes ERK, JNK, and p38 MAPK. Activation of these pathways increases the production of inflammatory cytokines, intensifying the inflammatory response, particularly in conditions like SAPHO syndrome (26–28). TNF-α impacts immune cells by promoting the recruitment of neutrophils and the activation of macrophages and T cells, thus enhancing the inflammatory milieu (**Figure 2**). TNF-α also affects bone cells, specifically osteoclasts and osteoblasts. TNF-α promotes osteoclast differentiation and activation, leading to increased bone resorption, while it inhibits osteoblast differentiation and function, impairing bone formation and repair. These dual actions on immune and bone cells underscore TNF-α’s pivotal role in the pathogenesis and progression of SAPHO syndrome, contributing to both inflammation and bone pathology (29–34).

### 4.2 IL-1β

**Figure 3:**
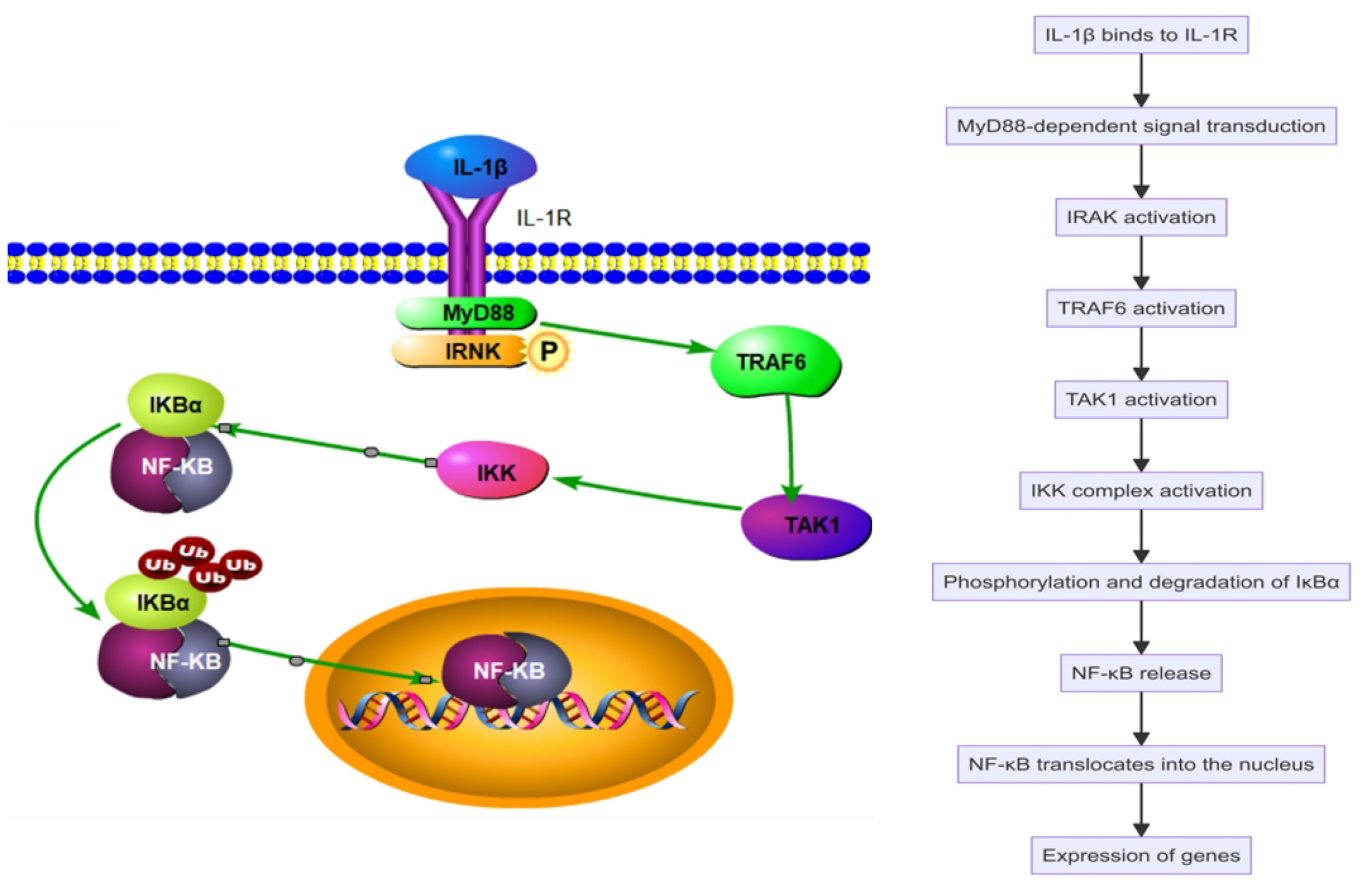
Interleukin-1 beta (IL-1β) activates the nuclear factor κB (NF-κB) pathway in the SAPHO syndrome.

Interleukin-1 beta (IL-1β), a key pro-inflammatory cytokine, plays a significant role in the pathogenesis of SAPHO syndrome. Produced primarily by activated macrophages and monocytes, IL-1β promotes the differentiation and activation of various immune cells, including macrophages, neutrophils, and T cells, enhancing the inflammatory response (**Figure 2**). IL-1β increases the production of other cytokines and chemokines, further amplifying the recruitment and activation of immune cells at inflammation sites (35–37). It activates multiple signaling pathways, including nuclear factor κB (NF-κB) and mitogen-activated protein kinase (MAPK), exacerbating SAPHO syndrome symptoms (38, 39). Upon binding to its specific receptor, IL-1R, IL-1β initiates MyD88-dependent signal transduction, activating downstream IRAK4 and IRAK1, which leads to the activation of TRAF6. TRAF6, a crucial signaling molecule, triggers TAK1, which subsequently activates the IKK complex and MAPKs. This activation sequence culminates in the phosphorylation and degradation of IκBα, releasing NF-κB to translocate into the nucleus and stimulate the expression of genes related to inflammation (**Figure 3**). Concurrently, activation of the MAPK pathway enhances the activity of the AP-1 transcription factor, intensifying the inflammatory response (40–45). Through these signaling pathways, IL-1β promotes the production of inflammatory mediators such as IL-6 and TNF-α and boosts the expression of chemokines and cell adhesion molecules that enhance the recruitment of inflammatory cells to inflammation sites. Additionally, in bone cells, IL-1β stimulates osteoclastogenesis, leading to increased bone resorption. IL-1β also inhibits osteoblast differentiation and function, impairing bone formation and repair. This dual action on immune and bone cells contributes to the chronic inflammation and bone pathology characteristic of SAPHO syndrome, exacerbating symptoms and disease progression (32, 42, 44, 46).

### 4.3 IL-6

**Figure 4:**
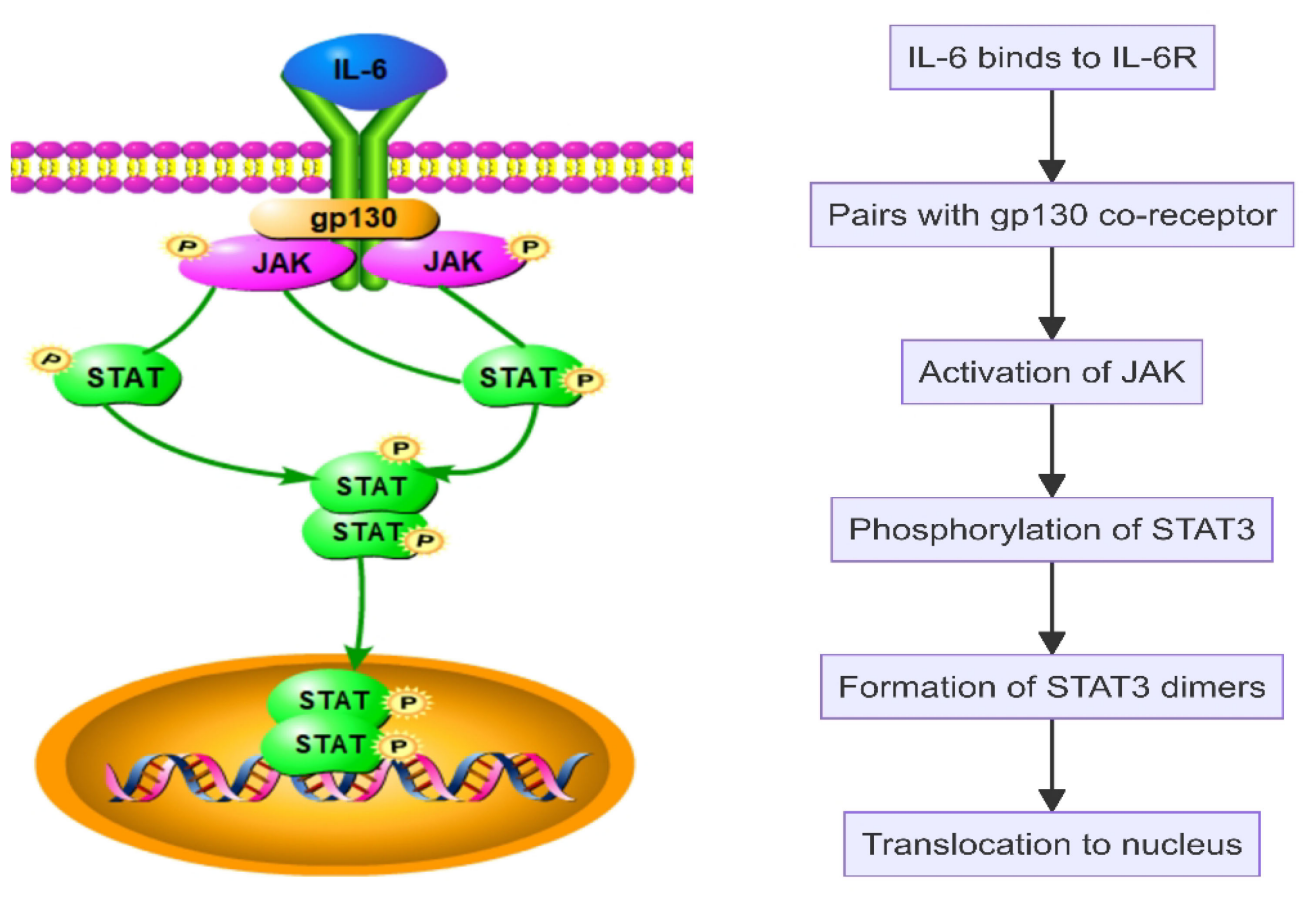
Interleukin-6 (IL-6) activates the JAK-STAT pathway in SAPHO syndrome.

Interleukin-6 (IL-6) plays a pivotal role in SAPHO syndrome by promoting the proliferation and differentiation of B cells and T cells, thereby enhancing the immune response (**Figure 2**). IL-6 initiates intracellular signaling by binding to its specific receptor, IL-6 receptor (IL-6R), and pairing with the gp130 co-receptor. This interaction activates Janus kinase (JAK), leading to the phosphorylation of signal transducer and activator of transcription 3 (STAT3). The phosphorylated STAT3 then forms dimers and translocates to the nucleus to stimulate gene expression related to inflammation, cell survival, and proliferation (**Figure 4**). Additionally, IL-6 engages the MAPK and PI3K-Akt signaling pathways, further influencing cellular metabolism and functions (46–49). In SAPHO syndrome, excessive IL-6 expression is strongly linked to the development of osteitis and skin lesions. It promotes bone resorption by enhancing osteoclast differentiation and activity while concurrently inhibiting osteoblast functions, thereby disrupting bone remodeling balance. This imbalance contributes to the typical bone lesions and pain in patients with SAPHO syndrome. Furthermore, IL-6 drives the differentiation of naive T cells into Th17 cells, a subset of T cells that produce IL-17, a potent pro-inflammatory cytokine. This differentiation process exacerbates the inflammatory response, contributing to the chronic inflammation observed in SAPHO syndrome. IL-6 also enhances the activation and survival of T cells, further sustaining the inflammatory environment (32, 46–48, 50).

### 4.4 IL-23 and IL-17

**Figure 5:**
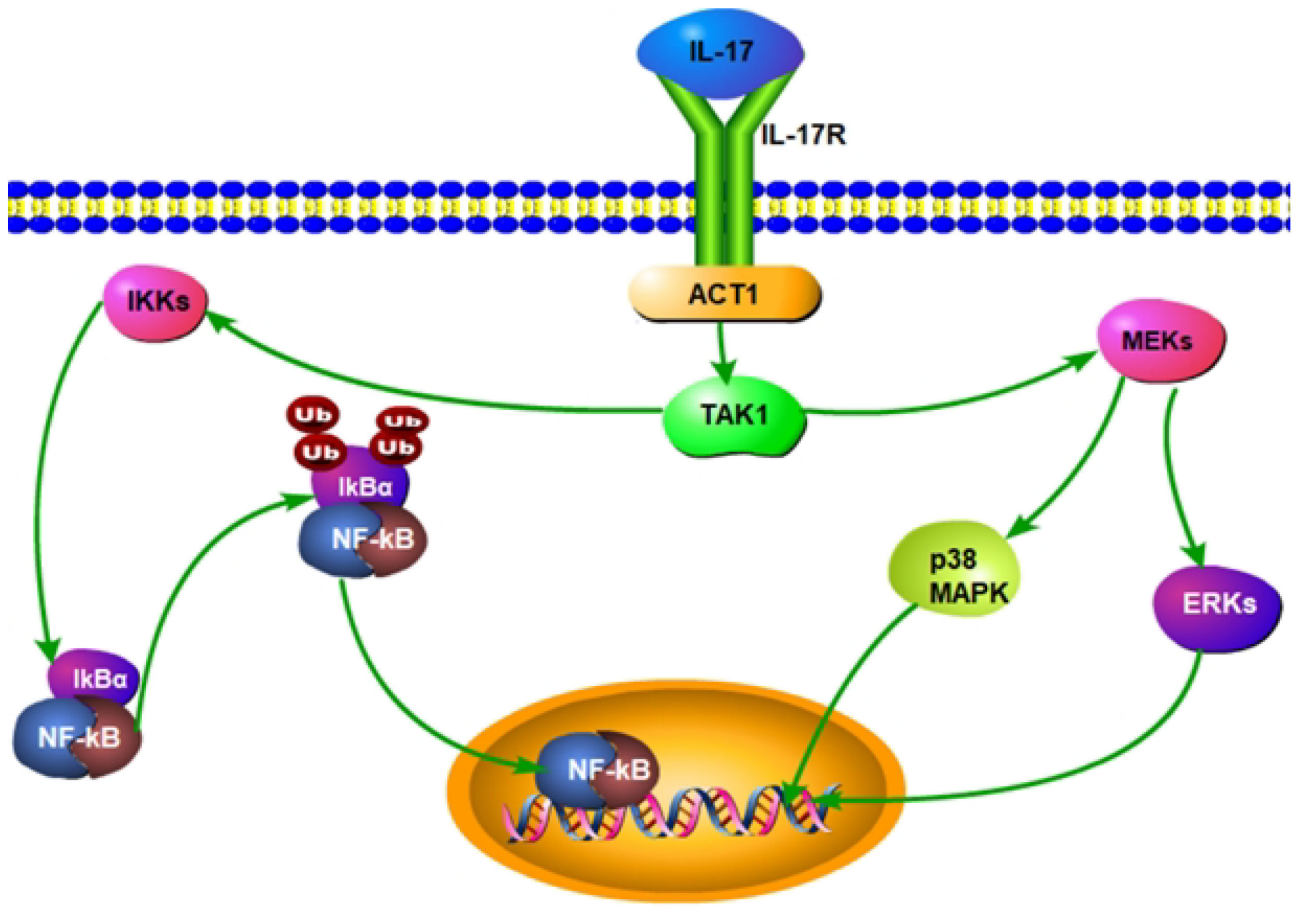
Interleukin-17 (IL-17) activates the nuclear factor κB (NF-κB) and Mitogen-Activated Protein Kinase(MAPK) pathways in SAPHO syndrome.

In the pathogenesis of SAPHO syndrome, interleukin-23 (IL-23) and interleukin-17 (IL-17) form a critical cytokine axis significantly influencing inflammation and bone lesions. IL-23, secreted by dendritic cells and macrophages, primarily functions to maintain and expand the Th17 cell population, the principal producers of IL-17 (51, 52). IL-23 also enhances the recruitment and activation of neutrophils and macrophages, contributing to a robust inflammatory response (**Figure 2**). IL-23 binds to its receptor complex, consisting of IL-23R and the co-receptor IL-12Rβ1, thereby activating the JAK/STAT signaling pathway, particularly STAT3. This activation promotes the differentiation and survival of Th17 cells. Th17 cells produce IL-17, which, upon binding to IL-17R, triggers downstream signaling pathways including NF-κB and MAPK, notably the p38 and ERK pathways (**Figure 5**). This process enhances the production of inflammatory mediators such as TNF-α, IL-1β, and IL-6, intensifying inflammation and tissue damage. IL-17 also enhances the recruitment and activation of neutrophils and macrophages, contributing to a robust inflammatory response (53–55). In SAPHO syndrome, dysregulation of the IL-23/IL-17 axis is closely linked to the distinctive symptoms of skin and bone lesions. In bone cells, IL-17 directly stimulates osteoclastogenesis, leading to increased bone resorption. IL-17 inhibits the differentiation and function of osteoblasts, impairing bone formation and repair. The combined effect of increased osteoclast activity and reduced osteoblast function results in the characteristic bone lesions of SAPHO syndrome. By affecting both immune and bone cells, IL-17 plays a pivotal role in the pathogenesis of SAPHO syndrome, driving the inflammation and bone pathology that are hallmarks of the disease (56–59).

### 4.5 IL-8

In SAPHO syndrome, IL-8 primarily recruits and activates neutrophils at inflammation sites. This chemokine not only attracts neutrophils but also enhances their adherence to endothelial cells, facilitating transendothelial migration. Once activated, neutrophils release enzymes and reactive oxygen species that contribute to inflammatory damage in SAPHO syndrome, evident in bone resorption and skin lesions (60). Beyond its chemotactic role, IL-8 also stimulates the production of other inflammatory cytokines. Specifically, IL-8 has been shown to upregulate the production of TNF-α and IL-1β, creating a feedback loop that amplifies the inflammatory response, which is crucial for sustaining chronic inflammation characteristic of SAPHO syndrome (17). IL-8 acts through multiple signaling pathways, primarily by binding to its receptors, CXCR1 and CXCR2, on neutrophils. This binding activates downstream signaling pathways including MAPK, NF-κB, and PI3K/Akt, leading to transcriptional activation of genes involved in inflammation, survival, and migration of immune cells, further contributing to the disease’s pathogenesis (61–63).

### 4.6 IL-18

Interleukin-18 (IL-18) plays a critical role in the inflammatory and pathogenic mechanisms of SAPHO syndrome, affecting both innate and adaptive immune responses. IL-18 enhances the activity of macrophages and dendritic cells, leading to increased production of pro-inflammatory cytokines such as TNF-α, IL-1β, and IL-6. This amplifies the inflammatory response, promoting chronic inflammation characteristic of SAPHO syndrome (14). IL-18 is key in activating Th1 and NK cell responses, enhancing the production of IFN-γ by these cells, thereby exacerbating the inflammatory response in SAPHO syndrome. Elevated levels of IL-18, observed in conditions with similar inflammatory profiles, suggest its involvement in sustained inflammatory processes in SAPHO syndrome (64, 65). IL-18 signals primarily through the IL-18 receptor (IL-18R), engaging the NF-κB signaling pathway, a central pathway in inflammatory responses. Activation of NF-κB leads to the transcription of various pro-inflammatory genes, amplifying the inflammatory response and contributing to tissue damage and bone remodeling observed in SAPHO syndrome (66, 67). Additionally, IL-18 activates crucial signaling molecules like MAPKs and the PI3K/AKT pathway, which are essential for propagating the inflammatory response. Besides its immune-stimulatory roles, IL-18 indirectly influences bone resorption in bone cells by promoting the production of RANKL, a key factor in osteoclast differentiation and activation. This results in increased osteoclast activity and bone resorption, contributing to the bone lesions typical of SAPHO syndrome. By affecting both immune and bone cells, IL-18 plays a critical role in the pathogenesis of SAPHO syndrome, exacerbating the inflammatory and bone remodeling processes (65, 67, 68).

### 4.7 IL-10

IL-10 is a potent anti-inflammatory cytokine known to inhibit the activation of various immune cells and the release of cytokines. In SAPHO syndrome, IL-10’s regulatory function potentially alleviates chronic inflammation and autoimmune responses by reducing the production of pro-inflammatory cytokines such as tumor necrosis factor α (TNF-α) and interleukin-1β (IL-1β) (18). Moreover, IL-10 promotes the differentiation of B cells and antibody production, enhancing immune regulation against infections and inflammation (69, 70). IL-10 activates STAT3 via the JAK-STAT signaling pathway, increasing the expression of anti-inflammatory genes such as SOCS3, which inhibits the activation of immune cells like macrophages and T cells, as well as the production of pro-inflammatory factors (71–73). Additionally, IL-10 enhances its anti-inflammatory effects through the PI3K/Akt pathway by activating mTOR, which inhibits NF-κB activation and the expression of downstream inflammatory genes (74).

### 4.8 TGF-β

**Figure 6:**
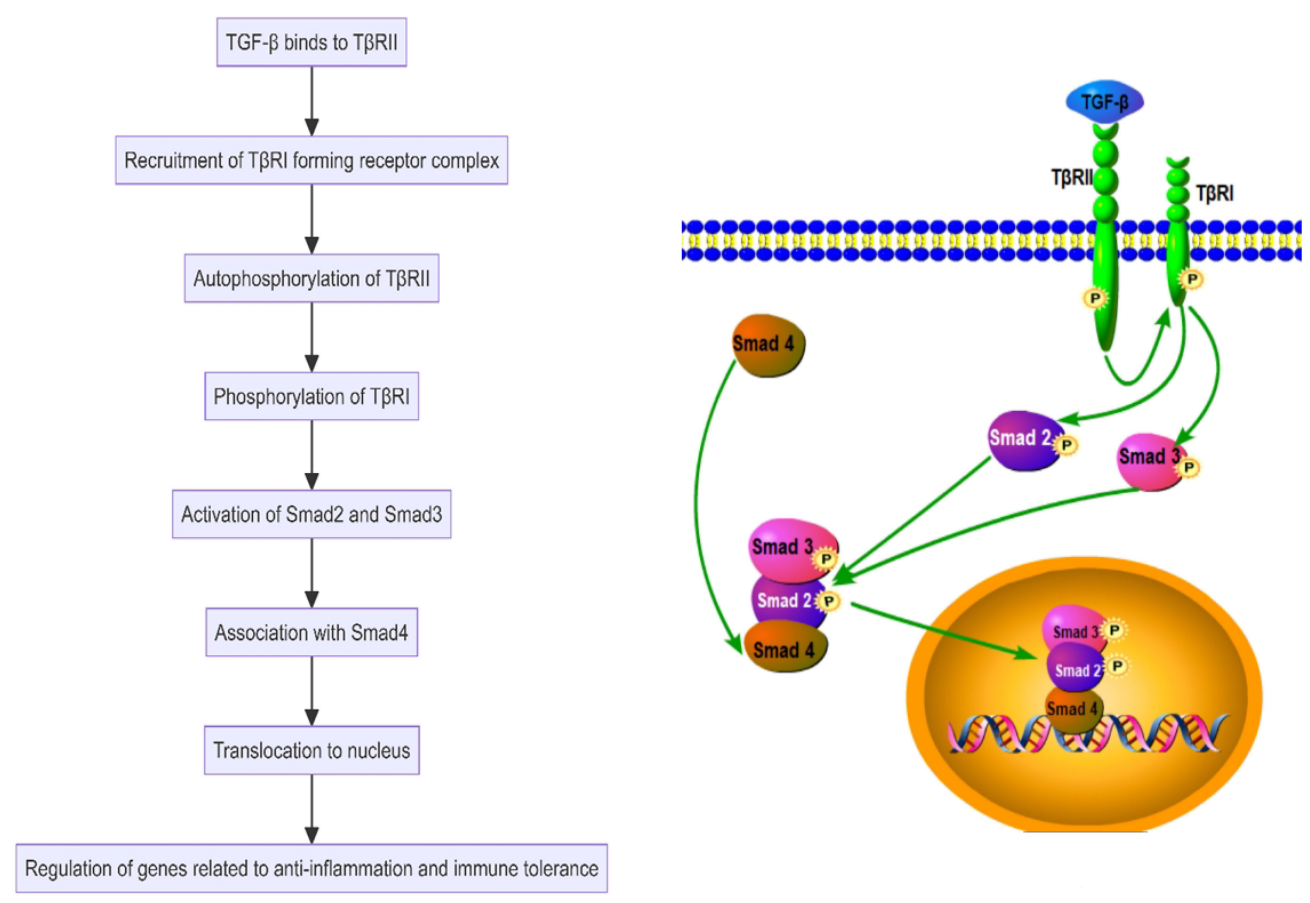
Transforming Growth Factor-beta (TGF-β) activates the Smad-dependent pathway in the SAPHO syndrome.

Transforming Growth Factor-beta (TGF-β), another key anti-inflammatory cytokine, helps maintain tissue homeostasis and immune tolerance. It reduces inflammation by inhibiting the activation of macrophages and T cells and supports immune balance by enhancing the differentiation and function of regulatory T cells, crucial for controlling autoimmune disease progression (75–78). TGF-β initially binds to the type II TGF-β receptor on the cell surface. This interaction facilitates the recruitment and binding of the type I TGF-β receptor, forming a receptor complex. Upon TGF-β binding, TβRII undergoes autophosphorylation and subsequently phosphorylates TβRI. This phosphorylation promotes the activation of Smad2 and Smad3, which then associate with Smad4 and translocate to the nucleus to regulate genes related to anti-inflammation and immune tolerance (79) (**Figure 6**). TGF-β also activates other signaling pathways such as MAPK and PI3K, further regulating cell survival, proliferation, and differentiation, thereby playing a multifaceted role in immune regulation (80, 81). In SAPHO syndrome, IL-10 and TGF-β collaboratively modulate immune cell activity and inflammatory response intensity, helping to control disease activity and progression. The functions of these cytokines extend beyond merely inhibiting inflammatory responses to include promoting tissue repair and regeneration, essential for the long-term management and treatment of patients with SAPHO syndrome (6, 18).

### 4.9 Cytokines and skin lesions connection

Numerous studies have investigated the connection between cytokines and skin lesions in SAPHO syndrome. Research has consistently demonstrated that cytokines, including TNF-α, IL-1, IL-6, IL-17, and IL-23, are abnormally expressed in the skin lesions of patients, often characterized by severe pustulosis and acne (18).

One particular study on patients with SAPHO syndrome showed that treatment with Secukinumab, an IL-17 inhibitor, significantly alleviated skin lesions and joint pain, indicating a crucial role of IL-17 in the associated skin manifestations. Furthermore, other research has revealed that anti-TNF-α treatments can provoke skin lesions in some patients, highlighting the influential role of TNF-α in these dermatological manifestations (56, 82).

Additionally, there is evidence suggesting that skin lesions in SAPHO syndrome may be linked to anomalies in the IL-23 pathway (83). For instance, paradoxical skin reactions have been observed in some patients following IL-17 inhibitor therapy, pointing to complex interactions between the IL-23 and IL-17 signaling pathways in affecting the syndrome’s dermatological features (84, 85).

In conclusion, skin lesions in SAPHO syndrome are intimately associated with the aberrant expression of various inflammatory cytokines, which intensify skin pathology by impacting inflammatory responses and immune regulatory mechanisms. Therefore, elucidating the roles and specific mechanisms of these cytokines in SAPHO syndrome is essential for developing precise therapeutic strategies.

## 5 The potential of cytokine targeted therapy for SAPHO syndrome

The diversity of clinical manifestations in SAPHO syndrome complicates its treatment. Current therapeutic strategies primarily focus on symptom relief, utilizing non-steroidal anti-inflammatory drugs (NSAIDs), corticosteroids, disease-modifying anti-rheumatic drugs (DMARDs), and bisphosphonates. Recent studies have underscored the effectiveness of bisphosphonates, such as zoledronic acid, in alleviating pain and reducing inflammatory lesions in patients with SAPHO syndrome. Cytokine-targeted therapy, aimed at specific inflammatory mediators integral to its pathogenesis, represents the cutting edge of treatments for SAPHO syndrome. IL-1 and TNF-α are considered critical to the inflammatory processes of the syndrome, with clinical trials of inhibitors like anakinra and infliximab demonstrating benefits in reducing symptoms and enhancing patient quality of life (10, 61, 86). Treatment of SAPHO syndrome continues to face significant challenges, particularly in target development. Recent studies have concentrated on identifying new biomarkers and therapies that influence the disease’s progression. For instance, the IL-23/Th 17 pathway has been recognized for its potential therapeutic value in SAPHO syndrome. Biologics targeting IL-23 and Th 17, such as Ustekinumab and Secukinumab, have proven somewhat effective, especially in ameliorating skin symptoms. Although the impact of these agents on osteoarticular symptoms remains uncertain, the research provides a scientific foundation for developing new therapeutic targets (56, 61, 85).

Furthermore, increasing evidence suggests a role for the microbiome in the pathogenesis of SAPHO syndrome. Propionibacterium acnes, for example, is thought to potentially exacerbate the condition by triggering host immune responses, offering a theoretical basis for the use of antibiotics, despite ongoing debates about their efficacy. Future research should continue to explore the interaction between the microbiome and the disease, aiming to develop targeted treatments based on this mechanism (15).

## 6 Conclusion

Cytokines are central to the pathogenesis of SAPHO syndrome, encompassing synovitis, acne, pustulosis, hyperostosis, and osteitis. Studies have indicated that pro-inflammatory cytokines, including tumor necrosis factor α (TNF-α), interleukin 1β (IL-1β), IL-6, and IL-8, are highly expressed in this disorder and contribute to heightened inflammatory responses. These cytokines drive the progression of SAPHO syndrome by influencing inflammation, inducing acute phase responses, and facilitating chemotaxis. Conversely, anti-inflammatory cytokines such as IL-10 and transforming growth factor-β (TGF-β) play protective roles by modulating immune responses and curbing excessive inflammation. Specifically, IL-10 mitigates symptoms by inhibiting the activities of TNF-α and IL-1, while TGF-β helps maintain tissue homeostasis and immune tolerance, thus decelerating the disease’s progression. These insights not only offer new therapeutic targets for SAPHO syndrome but also enhance understanding of its complex pathophysiological mechanisms.

Despite recent advances in cytokine research related to SAPHO syndrome, numerous unresolved issues persist. The precise pathogenesis, particularly the specific roles and interactions of various cytokines, remains elusive and necessitates further investigation. Moreover, while various biologics have demonstrated therapeutic promise, their clinical efficacy varies among individuals, highlighting the need for additional data to validate their long-term safety and effectiveness.

Future research should aim for a more comprehensive understanding of cytokine roles in SAPHO syndrome, especially their interplay and impact on disease progression. Additionally, there is a need to develop more personalized treatment strategies, potentially by customizing treatment plans according to individual cytokine expression profiles, to enhance therapeutic efficacy and safety.

## Author contributions

YY: Writing – original draft, Data curation. QC: Resources, Writing – review & editing. WZ: Conceptualization, Writing – original draft, Writing – review & editing.
